# Central sensitization in CRPS patients with widespread pain: a cross-sectional study

**DOI:** 10.1093/pm/pnad040

**Published:** 2023-03-22

**Authors:** Iara De Schoenmacker, Anna Mollo, Paulina Simonne Scheuren, Laura Sirucek, Florian Brunner, Petra Schweinhardt, Armin Curt, Jan Rosner, Michèle Hubli

**Affiliations:** Spinal Cord Injury Center, Balgrist University Hospital, University of Zurich, 8008 Zurich, Switzerland; Spinal Cord Injury Center, Balgrist University Hospital, University of Zurich, 8008 Zurich, Switzerland; Spinal Cord Injury Center, Balgrist University Hospital, University of Zurich, 8008 Zurich, Switzerland; Integrative Spinal Research, Department of Chiropractic Medicine, University Hospital Balgrist, University of Zurich, 8008 Zurich, Switzerland; Physical Medicine and Rheumatology, Balgrist University Hospital, 8008 Zurich, Switzerland; Integrative Spinal Research, Department of Chiropractic Medicine, University Hospital Balgrist, University of Zurich, 8008 Zurich, Switzerland; Alan Edward Center for Research on Pain, McGill University, Montreal, Quebec, Canada; Spinal Cord Injury Center, Balgrist University Hospital, University of Zurich, 8008 Zurich, Switzerland; Spinal Cord Injury Center, Balgrist University Hospital, University of Zurich, 8008 Zurich, Switzerland; Department of Neurology, University Hospital Bern, Inselspital, University of Bern, Bern, Switzerland; Danish Pain Research Center, Department of Clinical Medicine, Aarhus University, Aarhus, Denmark; Spinal Cord Injury Center, Balgrist University Hospital, University of Zurich, 8008 Zurich, Switzerland

**Keywords:** complex regional pain syndrome, spatial pain extent, central sensitization, quantitative sensory testing, temporal summation of pain

## Abstract

**Objective:**

Widespread pain hypersensitivity and enhanced temporal summation of pain (TSP) are commonly reported in patients with complex regional pain syndrome (CRPS) and discussed as proxies for central sensitization. This study aimed to directly relate such signs of neuronal hyperexcitability to the pain phenotype of CRPS patients.

**Methods:**

Twenty-one CRPS patients and 20 healthy controls (HC) were recruited. The pain phenotype including spatial pain extent (assessed in % body surface) and intensity were assessed and related to widespread pain hypersensitivity, TSP, and psychological factors. Quantitative sensory testing (QST) was performed in the affected, the contralateral and a remote (control) area.

**Results:**

CRPS patients showed decreased pressure pain thresholds in all tested areas (affected: t(34)  = 4.98, *P* < .001, contralateral: t(35) = 3.19, *P* = .005, control: t(31) = 2.65, *P* = .012). Additionally, patients showed increased TSP in the affected area (F(3,111) = 4.57, *P* = .009) compared to HC. TSP was even more enhanced in patients with a high compared to a low spatial pain extent (F(3,51) = 5.67, *P* = .008), suggesting pronounced spinal sensitization in patients with extended pain patterns. Furthermore, the spatial pain extent positively correlated with the Bath Body Perception Disturbance Scale (ρ = 0.491; *P* = .048).

**Conclusions:**

Overall, we provide evidence that the pain phenotype in CRPS, that is, spatial pain extent, might be related to sensitization mechanism within the central nociceptive system. This study points towards central neuronal excitability as a potential therapeutic target in patients with more widespread CRPS.

## Introduction

Complex regional pain syndrome (CRPS) is characterized by persisting pain disproportionate to the initiating event.^1^ Pain can be accompanied by a variety of other symptoms such as vasomotor, sudomotor, motor and trophic changes,^2^ and thus several pathomechanisms are thought to be involved in CRPS (for review see Bruehl 2015^3^).

CRPS patients commonly present with allodynia and hyperalgesia to mechanical stimuli which are not necessarily restricted to the affected area.^4^^,^^5^ Such hypersensitivities beyond the affected area are features of central sensitization^6^ and have previously been assessed contralaterally to the affected area or in a remote area such as the face.^4^^,^^5^^,^^7^^,^^8^ Central sensitization is defined as “increased responsiveness of nociceptive neurons in the central nervous system to their normal or subthreshold afferent input.”^9^ A surrogate marker of central sensitization is exaggerated temporal summation of pain (TSP),^10^ the human correlate of wind-up in animal studies, representing an increased excitability of dorsal horn neurons in the cat or rat spinal cord.^11–13^ A plethora of studies have reported increased TSP in chronic pain patients (eg, back pain or neuropathic pain after spinal cord injury) in affected as well as remote body areas.^14–18^ A further, rather clinical characteristic of sensitization within the central nervous system is a spread of pain beyond the expected anatomical region of pathology.^6^ This has been observed in various chronic pain conditions such as neuropathic pain after spinal cord injury,^19^ chronic pelvic pain,^20^ osteoarthritis,^21^ low back pain,^22^ and CRPS.^23^ In particular, CRPS patients showed three different patterns of pain distribution, that is, continuous spreading, independent spreading, and mirror-image spreading.^23^ The extent of the pain distribution has been related to other surrogate markers of central sensitization such as widespread pain hypersensitivities in patients with osteoarthritis.^21^ However, to our knowledge such a relationship has not yet been assessed in CRPS patients. Finally, psychological factors were associated with more severe pain, disability and poorer long-term recovery in CRPS.^24–27^ A review by Park and colleagues^28^ discussed that psychological factors may modulate the pain intensity of CRPS patients by altered neural circuits in the cortico-limbic system and increased nociceptive firing due to enhanced circulating catecholamine levels. Whether also the spatial pain extent is related to psychological factors remains unexplored. Therefore, the aim of this study was to investigate the relationship of different signs of central sensitization with the clinical pain phenotype in terms of the spatial pain extent in patients with CRPS. We hypothesized that an extended pain pattern will be associated with (1) widespread pain hypersensitivities and increased TSP, as well as (2) enhanced psychological distress in CRPS patients.

## Methods

### Study population

This study was part of a larger study including patients with CRPS, neuropathic pain after spinal cord injury, and low back pain, as well as age- and sex-matched healthy controls (HC), which were recruited between November 2019 and April 2022. The results presented in this manuscript are based on a consecutive sample of all patients with CRPS (N = 21) and 20 age-matched HC. CRPS patients were recruited at the Department of Physical Medicine and Rheumatology of the Balgrist University Hospital in Zurich, Switzerland, and diagnosed by an experienced rheumatologist (FB). All patients needed to fulfill the clinical Budapest Criteria^1^ at inclusion and had experienced pain for more than 3 months. Patients were excluded in case of neurological (eg, polyneuropathy, disk herniation), systemic (eg, autoimmune disease, diabetes) or clinically diagnosed psychiatric diseases or in case of pregnancy. HC had the same exclusion criteria with the addition of not having acute pain or a history of chronic pain (>3 months). Written informed consent was obtained from each participant and all experimental procedures were in accordance with the Declaration of Helsinki. The study was approved by the local ethics board “Kantonale Ethikkommission Zürich, KEK” (EK-04/2006, PB_2016-02051, clinicaltrial.gov number: NCT02138344).

### Study protocol

As previously stated, this study was part of a larger study, which comprised a comprehensive test battery (two visits of 3 hours each) including the evaluation of clinical pain characteristics, neurophysiological assessments, experimental pain paradigms, and psychological and pain questionnaires. The presented data in this manuscript are based on (1) psychological questionnaires (ie, Pain Catastrophizing Scale (PCS),^29^ the Hospital Anxiety and Depression Scale (HADS),^30^ and the Bath Body Perception Disturbance Scale (BBPDS)^31^); (2) assessment of the spatial pain extent, intensity and intake of pain medication; and (3) quantitative sensory testing (QST) including (4) TSP. The psychological questionnaires were completed electronically while QST (visit 1) and TSP (visit 2) were part of the test battery mentioned above.

### Psychological questionnaires

The PCS consists of 13 items, where participants have to reflect on past painful experiences and choose from different negative thoughts and feelings which are presented on a numerical scale from 0 (not at all) to 4 (all the time).^29^ The minimum and maximum scores of the PCS are therefore 0 and 52, respectively. The HADS consists of 14 items, where seven items particularly asses a general state of anxiety and seven items the general state of a depressive mood.^30^ The minimum and maximum scores for the items assessing anxiety and depression separately are 0 and 21, respectively. Scores less than seven indicate non-cases, scores between 8 and 10 indicate mild anxiety/depression, scores between 11 and 14 indicate moderate anxiety/depression, and scores between 15 and 21 indicate severe anxiety/depression. Finally, the BBPDS was developed to capture and quantify body perception disturbances in patients with CRPS.^32^ Here, some questions have to be rated on a scale from 0 (minimum) to 10 (maximum), whereas other questions are yes (1 point) or no (0 points) questions. The last question considers the description of the patients’ affected limb while keeping the eyes closed. The examiner draws the affected limb based on the patients’ description and evaluates the degree of distortion (0 = no distortion, 1= moderate distortion, 2 = severe distortion). The score of the BBPDS ranges from 0 to 57, where a higher score means more disturbed body perception.

### Pain characteristics and intake of pain medication

Pain characteristics included the spatial pain extent and the pain intensity. CRPS patients were asked to mark their painful areas by shading the corresponding area on two standardized body charts (dorsal and frontal view) on A4 papers. Here, only painful areas associated with CRPS were considered for further analysis. The borders of each area were marked manually by the investigator and run through a custom-made analysis software calculating the percentage of the marked body area (pixels) in relation to the entire body surface.^19^ Additionally, the average pain intensity of the last 4 weeks was specified. Moreover, to better describe the CRPS cohort, the duration of CRPS pain was specified. Further, the regular intake of pain medication was surveyed and classified into categories of anti-inflammatory and antirheumatic products (M01A), analgesics (opioidergic [N02A] and non-opioidergic [N02B]), anticonvulsants (N03), psycholeptics (N05), and psychoanaleptics (N06) according to the ATC/DDD classification by the World Health Organization [http://www.whocc.no/atc_ddd_index/].

### Measurement areas

The participants were measured in three different areas: their affected area, the contralateral homologous body site and in a remote, pain-free body area (control area). The contralateral and control area were measured to detect possible spinal and supraspinal sensitization, respectively.^10^ If the hand was affected, the contralateral shoulder was used as control area, whereas if the foot was affected, the contralateral hand was used as control area. If the shoulder was affected, no control area was measured. If multiple body regions were affected by CRPS or extended pain areas were present, the area with the momentarily highest pain rating within the affected area was measured. Individually matched HC were measured in the exact same areas as their corresponding CRPS patient.

### Quantitative sensory testing

The QST protocol was performed by trained experimenters based on the German Research Network on Neuropathic Pain (DFNS).^33^ It consists of the following measurements: cold detection threshold (CDT), warm detection threshold (WDT), thermal sensory limen (TSL, including paradoxical heat sensation [PHS]), cold pain threshold (CPT), heat pain threshold (HPT), mechanical detection threshold (MDT), mechanical pain threshold (MPT), stimulus-response function (SR-function, including mechanical pain sensitivity [MPS] and dynamic mechanical allodynia [DMA]), wind-up ratio (WUR), vibration detection threshold (VDT), and pressure pain threshold (PPT). Because the contralateral and the control area were primarily measured to examine central sensitization, only measures reflecting gain of function were assessed (ie, CPT, HPT, MPT, SR-function, WUR, and PPT). Due to time constraints, the SR-function assessed in the contralateral and control area consisted of only two (instead of five) stimulations of each pinprick (ie, 8 mN, 16 mN, 32 mN, 64 mN, 128 mN, and 256 mN) and dynamic light touch stimulation (ie, brush, cotton wool, and Q-tip). For the SR function, each stimulus was rated in terms of perceived pain on an NRS from 0 to 100 (0: no pain, 100: most pain tolerable). In accordance with the DFNS, the control area was always assessed first and the order of the affected and contralateral area was randomized. QST measures were z-transformed for each participant using the eQuiSTA software provided by the DFNS.

### Temporal summation of pain

TSP was assessed in response to twelve consecutive pinprick stimulations (MRC Systems, Heidelberg, Germany) applied at a frequency of 0.33 Hz only to the affected and control area. The order of measurement area was randomized. The stimulation intensity was set individually at an intensity of 4 on a numeric rating scale (NRS) from 0 to 10 (0: no pain, 10: most tolerable pain). The intensity of NRS 4 was assessed by a staircase method just before the TSP paradigm, with an upper limit of stimulation intensity at 512 mN. During the TSP protocol, participants were instructed to rate each stimulation on the NRS.

### Statistical analysis

All statistical tests were performed at an α level of 0.05 in R Studio statistical software (R version 4.1.2 for Windows). Missing data were excluded from further analysis (N/A).

#### Demographics, pain characteristics, and psychological factors

Demographics and questionnaire scores were tested for normality by a Shapiro Wilk test. The sex proportion within CRPS patients and HCs was compared by a Chi-squared test. Age, height, weight, and psychological factors (ie, HADS and PCS scores) were compared between CRPS patients and HCs using an unpaired *t*-test or Wilcoxon signed-rank test in the case of normal or non-normal distributed data, respectively. Additionally, the pain characteristics (ie, pain intensity and spatial pain extent) and psychological factors (ie, PCS, HADS, and BBPDS score) of CRPS patients were analysed using Pearson (for normally distributed data) or Spearman (for not normally distributed data) correlations with the corrplot() function from the corrplot package in R studio. Multiple comparison correction was applied using the Benjamini-Hochberg method.

#### Quantitative sensory testing

**Figure 1.**
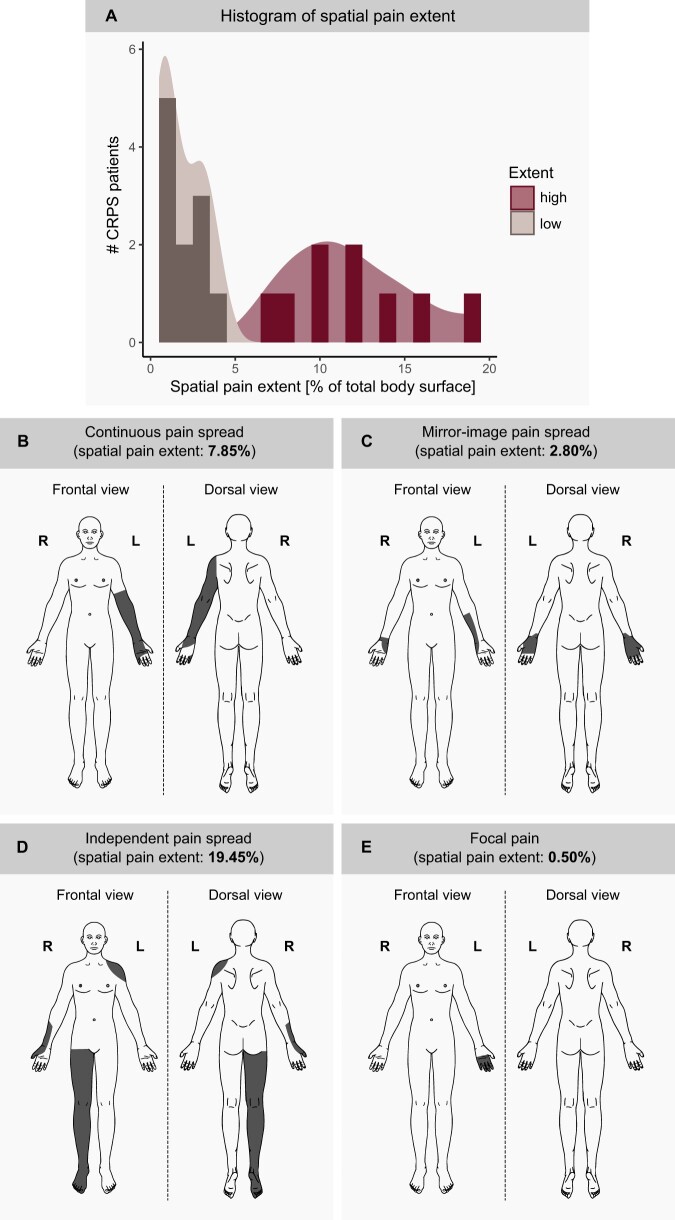
Spatial pain extent reported by the complex regional pain syndrome (CRPS) patients. (**A**) Histogram of the spatial pain extent. Illustrated are four different examples of CRPS patients with (**B**) continuous pain spread, (**C**) mirror-image pain spread, (**D**) independent pain spread, and (**E**) focal pain.

QST z-scores were tested for normality by the Shapiro Wilk test and compared between CRPS patients and HCs (unpaired *t*-test or Wilcoxon signed-rank test). The *t*-tests or Wilcoxen signed-rank tests were corrected for testing in multiple areas by the Benjamini-Hochberg method. Within the CRPS patients only, the z-scores of the different measurement areas were compared using a repeated measure ANOVA or Friedman test for either normally or not normally distributed data, respectively. Paired *t*-tests served as post-hoc test and were corrected for multiple comparisons using the Bonferroni method. Finally, the relationship between widespread pain hypersensitivities (significant gain of function in control area) and pain characteristics (ie, pain intensity and spatial pain extent) or psychological factors (ie, PCS, HADS, and BBPDS score) was investigated. The pain intensity and the psychological factors were normally distributed and thereby analyzed using Pearson correlations. The spatial pain extent was not normally distributed and after inspection of the descriptive statistics, two phenotypic subgroups were identified (high spatial pain extent >5% total body surface, N = 9; low spatial pain extent <5% total body surface, N = 11). A histogram of the spatial pain extent can be found in Figure 1A. Accordingly, the spatial pain extent was related to widespread pain hypersensitivities by comparing the two phenotypic subgroups using an unpaired *t*-test. Multiple testing within pain characteristics or psychological factors was corrected using the Benjamin-Hochberg method. In addition, a possible confounding effect of pain medication on widespread pain hypersensitivities was investigated by dividing patients into two subgroups according to whether they regularly take pain medication (yes/no). For this purpose, QST measures with a significant gain of function in the control area were compared between the pain medication subgroups by an unpaired *t*-test.

#### Temporal summation of pain

The differences in TSP between CRPS patients and HC was analyzed by general linear mixed models in order to keep the information of temporal aspect of TSP. The 12 stimulations of the TSP protocol were divided into four subblocks of three stimulations each to reduce the model’s dimensionality. The models were set up using the R package lme4^34^ with the averaged pain ratings across a subblock as dependent variable, the four stimulation subblocks as independent variable, and participant as random effect. Study cohort (CRPS or HC) was included as interaction effect with the stimulation subblock (subblock*cohort). The model *P* values was corrected for testing in multiple areas by the Benjamini-Hochberg method. Next, the association of pain characteristics (ie, pain intensity and spatial pain extent) and psychological factors (ie, PCS, HADS, and BBPDS score) with TSP was tested within the CRPS patients by including these factors as interaction effect with the stimulation subblock (subblock*pain-characteristic or subblock*psychological-factor). Pain intensity and psychological factors were included as numeric vector, whereas the spatial pain extent was included as phenotypic subgroup (high and low spatial pain extent). Testing within multiple areas and pain characteristics or psychological factors was corrected using the Benjamin-Hochberg method. Lastly, a potential confounding effect of pain medication on TSP was investigated by including the pain medication subgroup (yes/no) as factor into the model (subblock*medication). As previously, the *P* values were corrected for testing in multiple areas using the Benjamin-Hochberg method.

## Results

### Demographics, pain characteristics, and psychological factors

The demographics of the study population and information regarding psychological factors are shown in Table 1. CRPS patients had significantly higher HADS-anxiety (t(33) = 4.34, *P* < .001), HADS-depression (t(23) = 4.42, *P* < .001) and PCS scores (t(33) = 4.88, *P* < .001) compared to HC. CRPS-specific characteristics are presented in Table 2. Table S1 in the supplementary information section illustrates the intake of pain medication in detail. Examples of different pain patterns observed in CRPS patients are shown in Figure 1B–E. The correlation between pain characteristics and psychological factors of CRPS patients revealed that patients with a higher BBPDS scores presented with a larger spatial pain extent (ρ = 0.491, *P* = .048). The HADS and PCS, however, did not correlate with the spatial pain extent (HADS: ρ = .006, *P* = .979; PCS: ρ = −0.116, *P* = .740). Patients with a higher HADS and PCS score reported higher spontaneous pain intensities (HADS: r = 0.601, *P* = .021; PCS: r = 0.571, *P* = .021). The BBPDS score did not correlate with the pain intensity (r = 0.168, *P* = .701).

**Table 1.** Summary of the study population

	CRPS	HC	
	N = 21	N = 20	*P* value
Demographics			
Female [N] (%)	17 (81%)	14 (70%)	.414
Age [y]	44 (12)	45 (14)	.870
Height [cm]	170 (6)	170 (7)	.966
Weight [kg]	73 (14)	68 (12)	.300
Psychological questionnaires			
HADS anxiety [score]	8.0 (3.7)	3.6 (2.5)	**<.001**
HADS depression [score]	6.7 (4.8)	1.6 (1.5)	**<.001**
PCS [score]	22.2 (11.8)	7.2 (7.8)	**<.001**

Categorical data are presented as number of occurrences and relative proportion of the total data set. Continuous data are presented as mean (standard deviation).

CRPS = Complex Regional Pain Syndrome; HADS = Hospital Anxiety and Depression Scale; HC = Healthy Controls; PCS = Pain Catastrophizing Scale.

**Table 2.** CRPS-specific characteristics

CRPS-specific characteristics (N = 21)			
CRPS type I [N]	20	Continuous pain spreading [N]	11
CRPS type II [N]	1	Mirror-image pain spreading [N]	3
Etiology		Independent pain spreading [N]	2
Initial trauma [N]	16	Focal pain [N]	5
followed by surgery [N]	7	Spatial extent [% body surface]	4.1 (0.5–24.9)
Initial surgery [N]	5		
Affected hand [N]	13	Intensity [NRS]	5.5 (2.4)
Affected foot [N]	7		
Affected shoulder [N]	1	Duration [months]	35 (6–159)
Pain medication intake		BBPDS [score]	19.3 (8.1)
Yes [N]	14		
No [N]	7		

Normally distributed data are presented as mean (standard deviation) and not normally distributed data are presented as median (range).

BBPDS = Bath Body Perception Disturbance Scale; CRPS = Complex Regional Pain Syndrome.

### Difference in sensory profiles between patients with CRPS and HC

There were a few dropouts due to non-tolerance of the protocol encountered (full QST, WUR (N = 2), PPT (N = 1)). Furthermore, in two CRPS patients no control area could be assessed because it was either affected by pain (independent spreading pattern) or a frozen shoulder condition.

**Figure 2.**
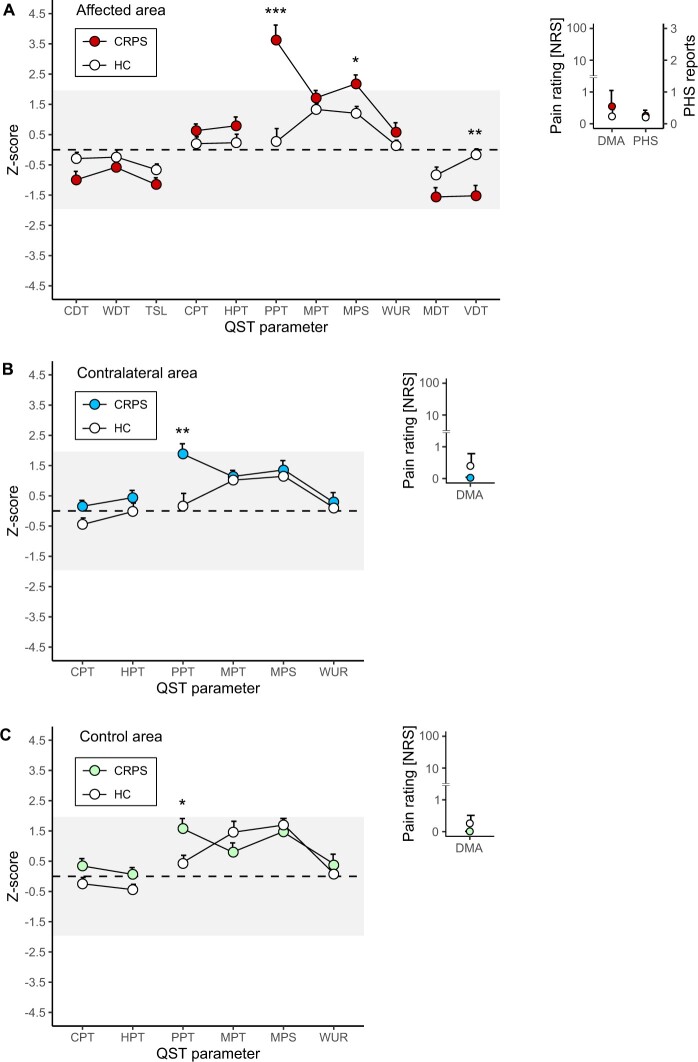
Sensory profiles of the affected, contralateral, and control area from complex regional pain syndrome (CRPS) patients and the corresponding areas in healthy controls (HC). Illustrated is the mean and standard error of measurement of z-scores and ±1.96SD (gray area). Sensory profile of the (**A**) affected, (**B**) contralateral, and (**C**) control area. CDT = cold detection threshold; CPT = cold pain threshold; DMA = dynamic mechanical allodynia; HPT = heat pain threshold; MDT = mechanical detection threshold; MPS = mechanical pain sensitivity; MPT = mechanical pain threshold; NRS = numeric rating scale; PHS = paradoxical heat sensation; PPT = pressure pain threshold; QST = quantitative sensory testing; TSL = thermal sensory limen; VDT = vibration detection threshold; WDT = warm detection threshold; WUR = wind-up ratio. *P < .05, **P < .01, ***P < .001.

CRPS patients showed a significant gain of function by means of decreased PPT and increased mechanical pain sensitivity (MPS) in the affected area compared to HC (PPT: t(34) = 4.98, *P* < .001, MPS: t(34) = 2.54, *P* = .048, Figure 2A). Additionally, CRPS patients showed significant loss of function in the affected area by means of an increased VDT compared to HC (t(30) = −3.37, *P* = .002). A gain of function in the contralateral (Figure 2B) as well as the control area (Figure 2C) of CRPS patients was measured by decreased PPT compared to HC (contralateral area: t(35) = 3.19, *P* = .005, control area: t(31) = 2.65, *P* = .012). There was no statistical difference between CRPS patients and HC for the remaining QST parameters (*P* > .05).

### QST across measurement areas in patients with CRPS

Within the CRPS cohort, PPT and MPT were different between the three measurement areas (PPT: F(2, 19) = 7.13, *P* = .009; MPT: F(2, 30) = 4.89, *P* = .015). Post-hoc analyses revealed that PPT was lower in the affected compared to the contralateral (t(17) = 2.99, *P* = .025) and control area (t(15) = 3.39, *P* = .012). In addition, MPT was lower in the affected compared to the control area (t(16) = 2.88, *P* = .033). There was no statistical difference between the stimulation areas for the remaining QST parameters (*P* > .05).

### Association between widespread hypersensitivity and pain characteristics or psychological factors

Widespread pain hypersensitivity, assessed by a significant gain of function (decrease in PPT) in the control area, did not correlate with pain intensity (r = 0.56, *P* = .066) or any of the psychological questionnaire scores (HADS: r = 0.52, *P* = .406; PCS: r = 0.42, *P* = .132; BBPDS: r = 0.06, *P* = .482). In addition, there was no difference in widespread pain hypersensitivity between CRPS patients with a high (>5%) compared to a low (<5%) spatial pain extent (t(14) = 0.68, *P* = .510). Finally, there was no difference in widespread pain hypersensitivity whether the patients regularly took pain medication or not (t(10) = 0.01, *P* = .993).

### Temporal summation of pain

One patient with CRPS did not tolerate the TSP protocol and had to be excluded from the TSP analysis. In addition, two patients did not have a control area as mentioned in the previous subsection.

**Figure 3.**
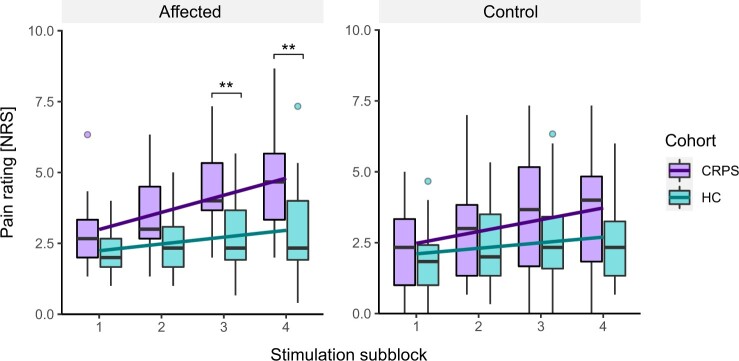
Temporal summation of pain in the affected (left) and control area (right) of CRPS patients (purple) and HC (turquoise). Illustrated is the mean pain rating (*y*-axis) of the stimulation subblocks (*x*-axis). The purple and turquoise lines illustrate the linear fit of the corresponding boxplots. The error bars illustrate the standard deviation. Additionally, post-hoc tests for the interaction effect between stimulation subblock and cohort are shown. CRPS = complex regional pain syndrome; HC = healthy controls; NRS = numeric rating scale. *P < .05, **P < .01, ***P < .001.

TSP was observed for both CRPS patients (affected area: F(3, 54) = 16.25, *P* < .001; control area: F(3, 54) = 13.30, *P* < .001) and HCs (affected area: F(3, 57) = 5.51, *P* = .002; control area: F(3, 57) = 5.62, *P* = .002). Figure 3 shows increased TSP in CRPS patients compared to HCs in the affected (F_subblock*cohort_(3, 111) = 4.57, *P* = .009), but not in the control area (F_subblock*cohort_(3, 111) = 2.08, *P* = .107). Post-hoc tests within the affected area are illustrated in Figure 3. There was no significant difference in TSP between the control and affected area of CRPS patients (F_subblock*area_(3, 126) = 0.38, *P* = .769).

### Relation between TSP and pain characteristics or psychological factors

**Figure 4.**
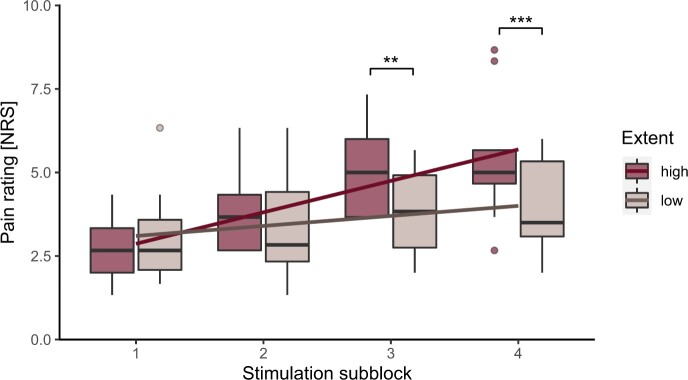
Temporal summation of pain (TSP) in the affected area of complex regional pain syndrome (CRPS) patients. The patient cohort is divided into two groups depending on their spatial pain extent (phenotypic subgroups). Patients with a high spatial pain extent are shown in red and patients with a low spatial pain extent in brown. Illustrated is the mean pain rating (*y*-axis) of the stimulation subblocks (*x*-axis). The burgundy and brown lines show the linear fit of the corresponding boxplots. The error bars illustrate the standard deviation. NRS = numeric rating scale. *P < .05, **P < .01, ***P < .001.

All results regarding the interaction effect between the TSP-subblock and pain characteristics or psychological factors are illustrated in Table 3. Importantly, CRPS patients with a high spatial pain extent (>5% of total body surface) showed more pronounced TSP in the affected area compared to patients with a low spatial pain extent (<5%) (Figure 4). Post-hoc tests within the affected area are illustrated in Figure 4. TSP tested in the control area was, however, not significantly different between the high and low spatial pain extent group. Pain intensity was neither related to TSP assessed in the affected area, nor to TSP assessed in the control area. Moreover, psychological factors did not significantly relate to both TSP measures. The intake of pain medication did not influence TSP regardless of the tested area.

**Table 3.** Interaction effects between TSP-subblock and other factors

Interaction effect with subblock	Affected Area		Control Area	
	F(3, 51)	*P* value	F(3, 51)	*P* value
Spatial pain extent	5.67	**.008**	1.78	.217
Pain intensity	0.97	.415	3.11	.068
HADS	0.39	.920	0.17	.920
PCS	0.53	.920	0.52	.920
BBPDS	0.25	.920	0.37	.920
Pain medication	0.49	.935	0.14	.935

## Discussion

The aim of this study was to better characterize the clinical pain phenotype of CRPS patients and relate it to signs of central sensitization and psychological distress. In line with previous work, this study provides additional evidence that central sensitization might contribute to the pathophysiology of CRPS^3^ indicated by mechanical hyperalgesia in remote pain-free body regions (widespread pain hypersensitivity) and increased TSP compared to HC. Strikingly, CRPS patients with an extended pain pattern showed more pronounced TSP in the affected area as well as higher disturbance of body perception compared to patients with a more focal pain phenotype.

### Signs of central and peripheral sensitization

Central sensitization is proposed to be a pathophysiological mechanism in CRPS^35^ and has previously been indicated by assessments of widespread pain hypersensitivities.^36^ Especially mechanical hyperalgesia in contralateral and remote areas was shown to be a hallmark of CRPS.^4^^,^^7^^,^^8^^,^^36^ Our finding of reduced PPT in the control area supports previous studies and indicates a general disturbance in central pain processing which might be due to hyperexcitable neurons in the central nervous system or deficient endogenous pain control. The latter was previously shown in patients with CRPS,^8^^,^^37^ and proposed as a main underlying mechanism of widespread pain hypersensitivity because of its spatially non-restricted nature.^38^ Alternatively, the observed widespread pain hypersensitivity might be induced by spinal sensitization through glial activation. Such widespread spinal sensitization was previously observed in rats with spinal cord injury, where astrocyte and microglia activation was present even 10 segments rostrally to the injury.^39^ Del Valle and colleagues supported this finding in a clinical CRPS case report.^40^ Post-mortem histology and immunochemistry were performed at different segmental levels of this chronic CRPS patient and revealed increased numbers of astrocytes and microglia in the dorsal horn of the CRPS patient compared to control subjects. Although the increase of microglia and astrocytes was most prominent at the segmental level of the affected area, it was present throughout the entire spinal cord.

Regardless of probable central hyperexcitability, a potential coexistence of peripheral sensitization, such as irritable primary nociceptive neurons, might additionally lower pain thresholds in the affected area.^41^ When comparing the sensory profiles of the different measurement areas within the CRPS patients we found lower MPTs in the affected compared to the contralateral or control area. This difference highlights a potential cumulative contribution of peripheral and central hypersensitivities in CRPS.

In addition to mechanical hyperalgesia, mechanical hypoesthesia was present in the affected area of CRPS patients (by means of reduced VDT). The combination of mechanical gain and loss of function was previously described in patients with CRPS.^42^ Previous literature suggested that such hypoesthesia results from cerebral reorganization due to continuous activation of the nociceptive neuraxis and is referred to as pain-induced hypoesthesia.^43^ The contribution of central plasticity to mechanical hypoesthesia in CRPS patients without any definable nerve lesion was previously supported by Pleger and colleagues.^44^ They showed reduced activity of the somatosensory cortex in response to electrical stimulation of the affected area which correlated with an increased two-point discrimination threshold. Additionally, neglect-like symptoms have been previously reported in CRPS patients,^45^ potentially contributing to the observed mechanical hypoesthesia in the affected limb.^46^

### Exaggerated TSP as sign of spinal sensitization

Increased TSP was seen in the affected area of CRPS compared to HC, supporting the hypothesis of a sensitized central nervous system in CRPS. TSP is considered to indicate increased excitability of second-order neurons located in the dorsal horn of the spinal cord.^11–13^ Consequently, we would argue that the observed increase in TSP in the affected area implies a hyperexcitability of these second-order neurons. Several potential mechanisms of spinal hyperexcitability have been previously discussed such as long-term potentiation of second-order neurons, disinhibition due to the loss of inhibitory interneurons, and glial-neuronal interactions (for review see Basbaum et al., 2009^47^). Previously, treatment with Ketamine (NMDA-receptor antagonist)^48^ and Baclofen (GABA-receptor agonist)^49^ have been shown to reduce CRPS specific symptoms by reducing the excitability of second-order neurons. These findings support the assumption that spinal hyperexcitability contribute to the pathophysiology of CRPS. When assessing the control area, however, TSP in CRPS patients was not increased compared to HC. On the one hand, this might be due to the greater variability in TSP when stimulating the control area of CRPS patients (SD = 2.20) compared to HC (SD = 1.59). On the other hand, a seminal preclinical study in rats has shown that dorsal horn neurons receiving input from deep muscle afferents (such as tested with PPT) are under more descending inhibitory control than spinal neurons receiving input from superficial cutaneous afferents.^50^ Therefore, it can be assumed that the increased hypersensitivity to painful pressure stimuli in the control area might better resemble a potential lack of descending inhibition, that is, net increase in spinal excitability, in CPRS patients than measures testing superficial cutaneous nociceptors (TSP).

### Association between experimental and clinical signs of central sensitization

Since some pain characteristics (eg, spatial pain extent) might indicate central sensitization,^38^ we hypothesized that these pain characteristics relate to psychophysical signs of central sensitization, for example, widespread pain hypersensitivity and increased TSP. In line with our hypothesis, patients with a high spatial pain extent had increased TSP in the affected area compared to patients with a low spatial pain extent. This strengthens the assumption that extended pain patterns in CRPS patients might occur due to hyperexcitable neurons within the central nervous system.^23^ The relationship between TSP and spatial pain extent was, however, not confirmed when studying TSP in the control area. This result might therefore further indicate that a large spatial pain extent is mainly a result of use-dependent plasticity within the spinal cord (ie, neurogenic inflammation, glial activation, and long term potentiation),^38^ which induces hyperexcitability within restricted body regions (ie, within the affected area).

Both spatial pain extent and pain intensity did not relate to widespread pain hypersensitivity (ie, reduced PPT in control area). Regarding spatial pain extent, these findings are in line with studies investigating patients with fibromyalgia and knee osteoarthritis.^16^^,^^51^ However, regarding pain intensity, these studies found that patients with higher pain intensity showed more signs of hypersensitivity.^16^^,^^51^ Although this tendency was also observed in our study, it could not be confirmed after correction for multiple comparisons. One possibility as to why no correlation was observed between the widespread pain hypersensitivity and pain intensity or spatial pain extent could be that underlying mechanisms are cumulative rather than congruent.

### Psychological factors are differently related to pain intensity and spatial pain extent

The spontaneous pain intensity correlated with psychological factors such as depression, anxiety, and pain catastrophizing whereas the spatial pain extent correlated with perceptual disturbances of the affected area. In line with these findings, previous systematic reviews in chronic pain conditions discussed the association between psychological factors and greater pain intensity.^52–55^ In particular with regard to patients with CRPS, Feldman and colleagues^27^ investigated in a prospective study the influence of depression and anxiety on pain intensity and vice versa. They found that pain led to increases in depression, anxiety, and anger the next day but also that depressed mood contributed to more pain the next day. Similarly, Farzad and colleagues^26^ found that psychological factors including, depression, anxiety, and pain catastrophizing were associated with greater pain intensity in patients with CRPS. However, the causality between psychological factors and pain intensity remains elusive.

Furthermore, a relationship between CRPS severity and body perception disturbances has previously been reported.^56^ These body perception disturbances were related to maladaptive cortical plasticity of the somatosensory cortex.^44^ Hence, such a cortical reorganization might contribute to the observed expansion of the spatial pain extent in our CRPS patients. This, however, is just speculative and should be investigated in further studies.

### Limitations

The sample size of patients with CRPS was rather small with a large variability in terms of pain characteristics (ie, pain intensity and spatial pain extent) and CRPS duration. On the one hand, this heterogeneity made it possible to investigate possible differences in terms of underlying mechanisms between these patients; on the other hand, it also limits the generalizability of the observed results. It would be of great interest to investigate whether these results are consistent in a larger patient sample reporting more homogeneous pain characteristics. Furthermore, the data presented are a subset of a larger test battery. The additional tests performed on the participants may have influenced the results presented by, for example, sensitizing the tested areas. Moreover, this study comes with the limitation that patients maintained their intake of pain medication during the period of study participation, potentially confounding our primary readouts. Nevertheless, we found no significant effect of pain medication on pain sensitivities assessed by QST or TSP. Finally, previous literature has mainly reported a pronounced ipsilateral spread of sensitization in patients with CRPS.^4^^,^^7^ Based on the measured control area in this manuscript, no conclusion can be drawn as to whether widespread sensitization mechanisms are more pronounced on the ipsilateral compared to the contralateral side of the affected area.

## Conclusion

To conclude, CRPS patients showed widespread pain hypersensitivity as means of decreased PPT in the control area and increased TSP in the affected area, corroborating previous studies indicate a potential presence of central sensitization in these patients. Most importantly, patients with high spatial pain extent showed increased TSP and body perception disturbances compared to patients with low spatial pain extent. Hence, the spatial pain extent itself might provide a clinically relevant measure for signs of sensitization mechanism within the central nervous system and may provide a marker for therapeutic success.

## Supplementary Material

pnad040_Supplementary_Data
