# Supplementary material for: Central sensitization in CRPS patients with widespread pain: a cross-sectional study
Source: Pain Med. 2023 Mar 22;24(8):974–84. doi: 10.1093/pm/pnad040 (PMC10391588; doi:10.1093/pm/pnad040)
Supplement: pnad040_Supplementary_Data [file pnad040_supplementary_data.docx]

# Supplementary information

Table S1: Regular pain medication intake of patients with CRPS.

| Medication group | N (total = 21) |
| --- | --- |
| Anti-inflammatory and anti-rheumatic products (M01A) | 4 |
| Analgesics  Only opioidergic (N02A)  Only non-opioidergic (N02B)  Both | 9  3  4  2 |
| Anticonvulsants (N03) | 6 |
| Psycholeptics (N05) | 1 |
| Psychoanaleptics (N06) | 8 |
